# The Mother

**Published:** 1855-03

**Authors:** 


THE MOTHER.—It has been truly said—The first being that rushes to the recollection of a soldier or a sailor, in his heart's difficulty, is his mother. She clings to his memory and his affection, in the midst of all forgetfulness and hardihood induced by a roving life. The last message he leaves is for her, his last whisper breathes her name. The mother, as she instills the lesson of piety and filial obligation into the heart of her infant son, should always feel that her labor is not in vain. She may drop into the grave—but she has left behind her influences that will work for her. The bow is broken, but the arrow is sped, and will do its office.

